# Intermedin attenuates renal fibrosis by induction of heme oxygenase-1 in rats with unilateral ureteral obstruction

**DOI:** 10.1186/s12882-017-0659-6

**Published:** 2017-07-11

**Authors:** Xi Qiao, Lihua Wang, Yanhong Wang, Xiaole Su, Yufeng Qiao, Yun Fan, Zhiqiang Peng

**Affiliations:** 1 Department of Nephrology, Second Hospital of Shanxi Medical University, Shanxi Kidney Disease Institute, 382, WuYi Road, Taiyuan, 030001 Shanxi People’s Republic of China; 2 Department of Microbiology and Immunology, Shanxi Medical University, 56, Xinjian Road, Taiyuan, 030001 Shanxi People’s Republic of China

**Keywords:** Intermedin, Renal, Fibrosis, Reactive oxygen species, Heme oxygenase-1

## Abstract

**Background:**

Intermedin [IMD, adrenomedullin-2 (ADM-2)] attenuates renal fibrosis by inhibition of oxidative stress. However, the precise mechanisms remain unknown. Heme oxygenase-1 (HO-1), an antioxidant agent, is associated with antifibrogenic effects. ADM is known to induce HO-1. Whether IMD has any effect on HO-1 is unclear. Herein, we determined whether the antifibrotic properties of IMD are mediated by induction of HO-1.

**Methods:**

Renal fibrosis was induced by unilateral ureteral obstruction (UUO) performed on male Wistar rats. Rat proximal tubular epithelial cell line (NRK-52E) was exposed to rhTGF-β1 (10 ng/ml) to establish an in vitro model of epithelial-mesenchymal transition (EMT). IMD was over-expressed in vivo and in vitro using the vector pcDNA3.1-IMD. Zinc protoporphyrin (ZnPP) was used to block HO-1 enzymatic activity. IMD effects on HO-1 expression in the obstructed kidney of UUO rat and in TGF-β1-stimulated NRK-52E were analyzed by real-time RT-PCR, Western blotting or immunohistochemistry. HO activity in the obstructed kidney, contralateral kidney of UUO rat and NRK-52E was examined by measuring bilirubin production. Renal fibrosis was determined by Masson trichrome staining and collagen I expression. Macrophage infiltration and IL-6 expression were evaluated using immunohistochemical analysis. In vivo and in vitro EMT was assessed by measuring α-smooth muscle actin (α-SMA) and E-cadherin expression using Western blotting or immunofluorescence, respectively.

**Results:**

HO-1 expression and HO activity were increased in IMD-treated UUO kidneys or NRK-52E. The obstructed kidneys of UUO rats demonstrated significant interstitial fibrosis on day 7 after operation. In contrast, kidneys that were treated with IMD gene transfer exhibited minimal interstitial fibrosis. The obstructed kidneys of UUO rats also had greater macrophage infiltration and IL-6 expression. IMD restrained infiltration of macrophages and expression of IL-6 in UUO kidneys. The degree of EMT was extensive in obstructed kidneys of UUO rats as indicated by decreased expression of E-cadherin and increased expression of α-SMA. In vitro studies using NRK-52E confirmed these observations. EMT was suppressed by IMD gene delivery. However, all of the above beneficial effects of IMD were eliminated by ZnPP, an inhibitor of HO enzyme activity.

**Conclusion:**

This study demonstrates that IMD attenuates renal fibrosis by induction of HO-1.

**Electronic supplementary material:**

The online version of this article (doi:10.1186/s12882-017-0659-6) contains supplementary material, which is available to authorized users.

## Background

Chronic kidney disease (CKD) is a major health problem and its incidence and prevalence are increasing worldwide [1]. Regardless of disease etiology, fibrosis is a final common pathogenic process for CKD leading to end stage renal diseases (ESRD) [2]. Renal fibrosis is characterized by excessive accumulation of extracellular matrix (ECM) in the tubulointerstitium. Reactive oxygen species (ROS) play an important pathogenic role in the development of renal fibrosis by inducing epithelial-mesenchymal transition (EMT) of tubular epithelial cells and by inducing macrophage infiltration [3–5]. Intermedin [IMD, also known as adrenomedullin-2 (ADM-2)] is a novel member of the calcitonin/calcitonin gene-related peptide family [6]. Our previous study demonstrated that kidney-specific IMD gene transfer significantly ameliorated unilateral ureteral obstruction (UUO)-mediated fibronectin up-regulation and the fibrotic changes, and the anti-fibrotic effect is achieved by inhibition of oxidative stress [7]. However, the precise mechanisms remain unknown.

Heme oxygenase (HO) is the rate-limiting enzyme in the degradation of heme [8]. It converts heme to biliverdin via a reaction that produces carbon monoxide and liberates iron [9]. There are two isozymes for HO, an inducible form (HO-1) and a constitutive one (HO-2) [10]. HO-1 is strongly induced by oxidative stress and shows cytoprotective effects by the anti-inflammatory, anti-apoptotic, and anti-proliferative actions [11]. Its expression is upregulated in renal fibrosis [12], and furthermore, recent study demonstrated that induction of HO-1 prevented renal fibrosis induced by UUO [13].

ADM is reported to induce HO-1 gene expression in rat vascular smooth muscle cells [14]. Whether IMD has any effect on HO-1 has not been investigated yet. Since IMD and ADM have similar biological actions, we hypothesized that IMD can also induce HO-1 expression and that may explain its anti-oxidant and anti-fibrotic effects. To test this hypothesis, we investigated the effects of IMD overexpression on HO-1 expression and HO activity in the obstructed kidney of UUO rat and in rhTGF-β1-stimulated rat renal proximal tubular epithelial cells (NRK-52E). To evaluate the role of HO-1 induction in the protective effects of IMD on renal fibrosis, zinc protoporphyrin (ZnPP), an HO activity inhibitor [15], was used. Furthermore, NRK-52E were analyzed for the effect of IMD on EMT.

## Methods

### Animals and UUO-induced renal fibrosis model

Ethics Committee for Animals of Shanxi Medical University approved all animal protocols. Male Wistar rats (180 to 200 g) were purchased from the Experimental Animal Center of Shanxi Medical University (Taiyuan, China) and maintained in a specific pathogen-free environment in our facility. All animal were fed with standard chow and had free access to water. Renal fibrosis was induced by ligation of left ureter, UUO, as we previously reported [7]. Briefly, rats were anaesthetized, laparotomy performed, and the left ureter identified and ligated at two points along the ureter. Sham-operated rats underwent the same surgical procedure except for the ureter ligation.

### Ultrasound-mediated gene delivery into the kidney

The eukaryotic expression plasmid pcDNA3.1-IMD containing fulllength complementary DNA (cDNA) sequence of rat IMD was successfully constructed in our previous study [16]. Before the ureter was obstructed, pcDNA3.1-IMD plasmid or control empty vector was transfected into the left kidney via the renal artery using an ultrasound-microbubble mediated system as we previously described [16]. Rats were killed at 7 days after UUO. The transfection rate was detected by quantitative RT-PCR and Western blot analysis, respectively.

### Experiment design

#### Animal study

Rats were randomly divided into the following five groups: sham, UUO, UUO + empty plasmid, UUO + IMD and UUO + IMD + ZnPP. Rats in UUO + empty plasmid, UUO + IMD and UUO + IMD + ZnPP groups were treated with ultrasound as described above before UUO operation. In UUO + IMD + ZnPP group, ZnPP (50 μg/kg body wt, Sigma-Aldrich, St. Louis, MO, prepared in the dark) was administered intraperitoneally with a single dose 48 h before UUO operation as previously reported [17]. Animals were killed at 7 days after UUO. Obstructed kidneys and contralateral kidneys were harvested for further analysis.

#### Cell culture and treatments

Rat proximal tubular epithelial cell line (NRK-52E) was obtained from the Cell Bank of the Chinese Academy of Sciences (Shanghai, China). Cells were cultured in DMEM/Ham’s F-12 (2:1) containing with 10% fetal bovine serum and incubated at 37 °C, room air with 5% CO_2_. Cells were then cultured in fibronectin pre-coated flasks until they reached approximately 80% confluence. The over-expression of IMD in the NRK-52E cells that were transfected with pcDNA3.1-IMD was accomplished according to the methods described in our previous study [18]. The model of EMT in the NRK-52E cells was established by stimulating with recombinant human TGF-β1 (rhTGF-β1, 10 ng/ml, Gibco) for 72 h. The cells were assigned to 1 of 5 groups: control (untreated NRK-52E cells); TGF-β1 (NRK-52E cells were incubated with rhTGF-β1); TGF-β1 + empty vector (NRK-52E cells were transfected with the empty plasmid pcDNA3.1 and incubated with rhTGF-β1); TGF-β1 + IMD (NRK-52E cells were transfected with pcDNA3.1-IMD and incubated with rhTGF-β1); and TGF-β1 + IMD+ ZnPP [NRK-52E cells were transfected with pcDNA3.1-IMD and incubated with rhTGF-β1 plus 2 μM ZnPP (prepared in the dark)].

### Quantitative RT-PCR

Total renal RNA was extracted with Trizol reagent (Invitrogen, Carlsbad, CA, USA), and then subjected to RT using a first-strand cDNA synthesis system (TAKARA, Dalian, China). Quantitative RT-PCR amplifications were performed in triplicate using the SYBR Green I assay and were carried out using Strategene M3000 Sequence Detection System (Stratagene, Santa Clara, CA, USA). Specific primers were used for IMD (sense: 5′-GGCCCAGTTGCTGATGGT-3′ and antisense: 5′-TGCCCGGGAGCAGGTA-3′), HO-1 (sense: 5′-AGGTGCACATCCGTGCAGAG -3′ and antisense: 5′-CTTCCAGGGCCGTATAGATATGGTA-3′) and β-actin (sense: 5′-CCCATCTATGAGGGTTACGC-3′ and antisense: 5′-TTTAATGTCACGCAC GATTTC-3′). The reaction was carried out in a 96-well plate in 25 μL reactions containing 2 × SYBR Green Master mix (TAKARA, Dalian, China)—2 pmol each of sense and antisense primer and the conditions were 95 °C for 2 min, followed by 95 °C for 10 s, 60 °C for 15 s, for 40 cycles. In each assay, a standard curve was determined concurrently with examined samples. Gene expression was quantified using a medication of the 2-ΔΔct method. The amount of PCR products was normalized to the level of β-actin to determine the relative expression ratio for each mRNA.

### Western blot analysis

Analysis was carried out as previous described [16]. Immunoblot analysis was performed with rabbit polyclonal anti-IMD (Beijing Biosynthesis Biotechnology, Beijing, China), goat polyclonal anti-HO-1 (Santa Cruz Biotechnology), mouse monoclonal anti-collagen I (Col-I, Santa Cruz Biotechnology), mouse monoclonal anti-α-SMA (Sigma-Aldrich), or rabbit moloclonal anti-E-cadherin (Abcam, Cambridge, UK) and then peroxidase conjugated AffiniPure secondary antibodies (Santa Cruz Biotechnology), respectively. The ECL Western blotting system (Santa Cruz Biotechnology) was used for detection. Rabbit polyclonal anti-β-actin (Santa Cruz Biotechnology) was used as the control for each sample.

#### HO enzymatic activity assay

HO activity was estimated by determining the level of bilirubin generated in isolated microsomes as described earlier [19]. In brief, homogenized samples from the obstructed kidneys, contralateral kidneys or cultured NRK-52E cells were incubated in medium consisting of heme (0.25 mmol/L), rat liver cytosol (5 mg/mL) as a source of biliverdin reductase, MgCl_2_(1 mmol/L), glucose-6-phosphate dehydrogenase (1 unit), glucose-6-phosphate (0.8 mmol/L) and NADPH (0.8 mmol/L) in 0.5 mL of 0.1 mol/L phosphate buffer saline (pH 7.4) for 1 h at 37 °C in the dark. Reactions were terminated by ice and then the bilirubin product was extracted with chloroform. Absorbance was measured using a spectrometer at wavelengths 464 and 530 nm. HO activity was calculated by the difference in absorbance between the two wavelengths, and expressed as picomoles of bilirubin per milligram of protein per h.

### Immunohistochemical analysis

Rat kidneys were fixed in 10% formaldehyde and embedded in paraffin. Paraffin sections (4 μm) were mounted on glass slides, deparaffinized in xylene, and rehydrated in ethanol with increasing concentrations of water. The rehydrated sections were pretreated with 3% H_2_O_2_ for 10 min at room temperature to block the endogenous peroxidase. After boiling in antigen retrieval solution (1 mmol/L tris-HCl, 0.1 mmol/L EDTA, pH = 8.0) for 10 min at high power in a microwave oven, the sections were incubated overnight at 4 °C with primary goat polyclonal anti-HO-1 (1:200, Santa Cruz Biotechnology), mouse monoclonal anti-ED-1 (1:200, Santa Cruz Biotechnology) or mouse moloclonal anti-IL-6 (1:200, Abcam, Cambridge, UK). This was followed by biotinylated secondary antibodies (Santa Cruz Biotechnology) and finally by avidin conjugated horseradish peroxidase. All slides were counterstained with haematoxylin. HO-1 or IL-6 positive stained areas were assessed and expressed as integrated optical density (IOD) and area. Interstitial macrophages were expressed as the percentage of ED-1 positive interstitial area. Three sections of each rat kidney were measured, and 10 random fields were chosen and calculated under magnification of 400×. The IOD and positive area were acquired by the Image-Pro Plus 6.0 program (Media Cybernetics, Bethesda, MD, USA).

### Histological examinations

Renal histological changes were assessed at days 7 after UUO. Paraffin-embedded transverse kidney slices were sectioned at 4 μm. For analyzing the degree of tubulointerstitial collagen deposition, sections were stained with Masson trichrome. Twenty cortical tubulointerstitial fields that were randomly selected at ×200 magnifications were assessed in each rat, and the density of trichrome positive signals was analyzed using Image-Pro Plus 6.0 program (Media Cybernetics, Bethesda, MD, USA). Briefly, the tonality of the fibrosis area (blue) was determined with control sections as reference. The number of pixels with the predetermined color of tone was counted in each section, and then automatically converted into dimensions.

### Immunofluorescence

NRK-52E monolayers were fixed in methanol (20 min, −20 °C), and blocked with 2% bovine serum albumin (BSA) in PBS (1 h, room temperature). Cells were then incubated with primary antibodies against epithelial cell marker rabbit moloclonal anti-E-cadherin (1:50, Abcam, Cambridge, UK) and mesenchymal marker mouse monoclonal anti-α-SMA (1:100; Sigma-Aldrich) with 2% BSA overnight at 4 °C). Secondary fluorescent conjugated anti-rabbit Alexa Fluor®647 (1:400; Invitrogen) was used for E-cadherin and anti-mouse Alexa Fluor®546 (1:400; Invitrogen) was used for α-SMA for 40 min at room temperature in dark. Slides were mounted using ProLong® Gold Antifade reagent with DAPI (Life technologies). Images were photographed using Olympus FV1000 Confocal microscope at ×40 magnification.

### Statistical analysis

The data are expressed as means ± standard deviation (SD). Significant differences between groups were assessed by one-way ANOVA with Student-Newman-Keuls post hoc tests. *P* values <0.05 were considered statistically significant.

## Results

### IMD is transfected into the kidney or into NRK-52E cells successfully

**Fig. 1:**
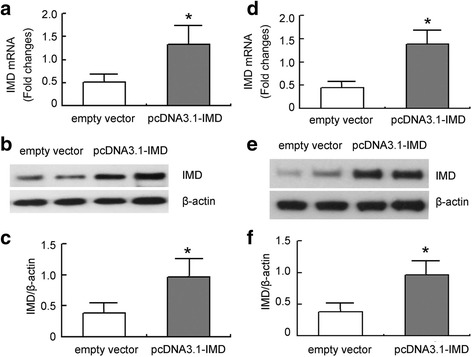
The transfection efficiency of IMD by ultrasound-mediated gene delivery into the kidney and by FuGENE HD into NRK-52E cells. **a** IMD mRNA expression measured by quantitative RT-PCR in the obstructed kidney of UUO rats. **b** Representative IMD protein expression measured by Western blot in the obstructed kidney of UUO rats. **c** Densitometric quantifications of band intensities from Western blot for IMD/β-actin in the obstructed kidney of UUO rats. **d** IMD mRNA expression measured by quantitative RT-PCR in NRK-52E cells. **e** Representative IMD protein expression measured by Western blot in NRK-52E cells. **f** Densitometric quantifications of band intensities from Western blot for IMD/β-actin in NRK-52E cells. Data in *bar graphs* are means ± SD, *n* = 6. ∗ *P* < 0.05 vs. empty vector group

The transfection efficiency of IMD by ultrasound mediated gene delivery into the Kidney in vivo and by FuGENE HD into NRK-52E cells in vitro was examined by quantitative RT-PCR and Western blot analysis. We showed that after 7 days of transfection, obstructed kidneys from rats treated with pcDNA3.1-IMD plasmid exhibited significant increase in IMD expression compared with that from rats treated with control empty vector (Fig. 1a–c). Similarly, the expression of IMD in NRK-52E cells transfected with pcDNA3.1-IMD plasmid was much high than in controls, indicating that IMD was transfected into the kidney or NRK-52E successfully (Fig. 1d–f).

### HO-1 is upregulated and HO activity is increased in the obstructed kidney of UUO rats, and is further induced by IMD gene transfer

**Fig. 2:**
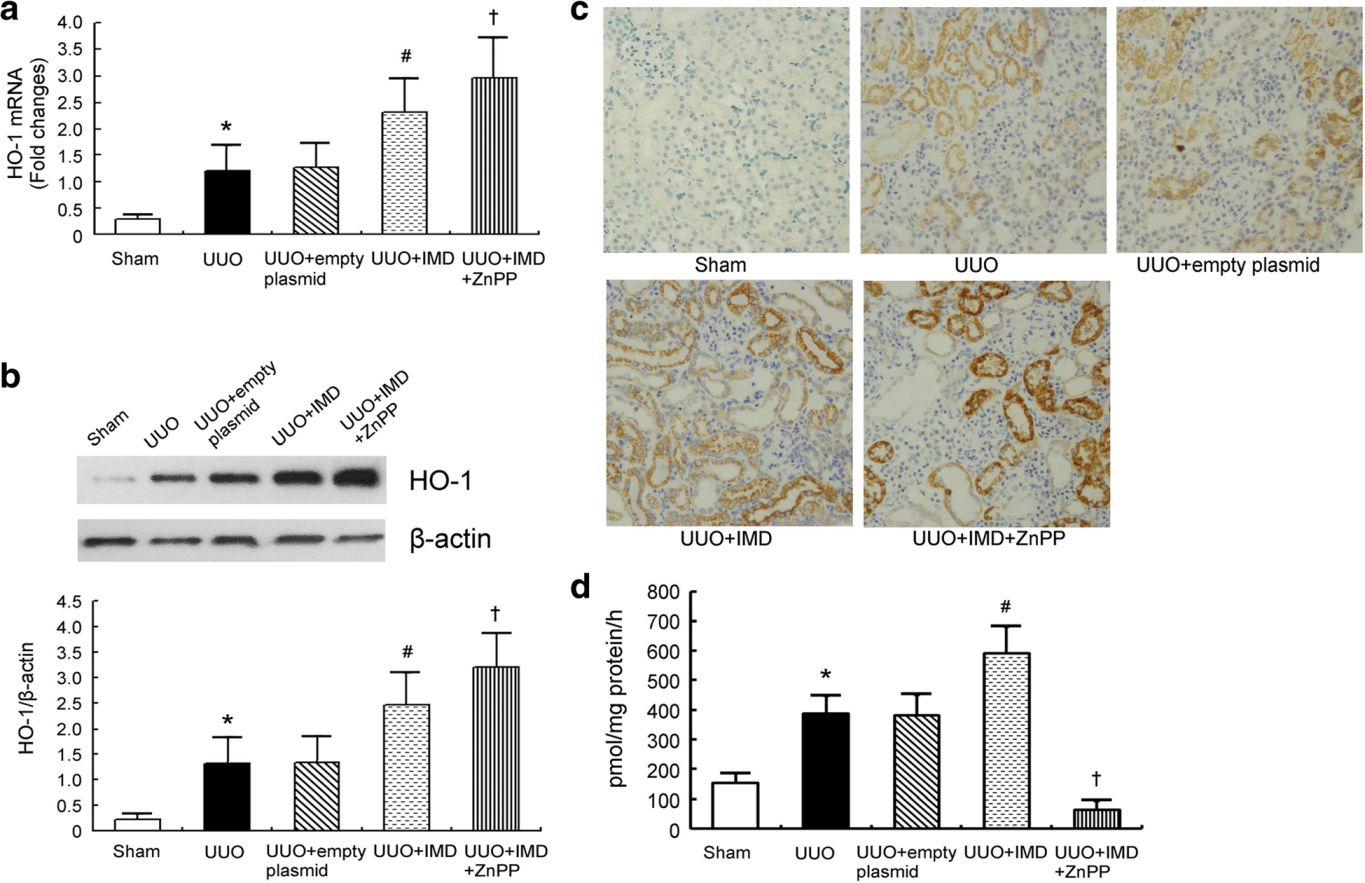
HO-1 expressions in the obstructed kidney of UUO rats. **a** Analysis of HO-1 mRNA levels in the obstructed kidneys from rats after UUO. **b** Respective Western blot analysis and quantitation of HO-1 protein levels in the obstructed kidneys from rats after UUO. **c** Representative immunohistochemical images for HO-1 in obstructed kidneys sections on day 7 after UUO. (Magnification, ×200). **d** HO activity in the obstructed kidneys of UUO rats. Data in *bar graphs* are means ± SD, *n* = 6.∗ *P* < 0.05 vs. sham control group; # *P* < 0.05 vs. UUO group; † *P* < 0.05 vs. UUO + IMD group

HO-1 mRNA and protein expression was very low in the sham-operated kidneys, whereas there was a significant increase in HO-1 mRNA and protein abundance in the obstructed kidney after UUO. IMD gene transfer further increased HO-1 expressions in the obstructed kidneys, while the control empty plasmid had no effect on HO-1 levels. Interestingly, the levels of HO-1 mRNA and protein were also increased in UUO rats with IMD and ZnPP combined treatment (Fig. 2a and b). The result was in line with previous report [17].

Localization of HO-1 protein in the obstructed kidneys was examined by immunohistochemical analysis. We showed that HO-1 immunoreactivity was increased in the obstructed kidneys of UUO rats. Positive staining of HO-1 protein was mainly observed in tubular cells. HO-1 expression was further enhanced by IMD gene delivery. Immunohistochemical studies also demonstrated an upregulation of HO-1 with additional ZnPP treatment. Control empty plasmid did not affect the expression level of HO-1(Fig. 2c).

HO activity was significantly increased in UUO kidneys compared to sham controls. Pretreatment with IMD further elevated HO enzymatic activity in the obstructed kidneys of UUO animals. Administration of ZnPP abrogated IMD action on HO activity. Control empty plasmid had no effect on HO activity (Fig. 2d). We also evaluated the effect of IMD on HO activity of the contralateral kidneys of UUO rats, and didn’t find any difference between IMD treated UUO rats and untreated UUO controls (Additional file 1: Figure S1).

### IMD gene delivery induces HO-1 expression and HO activity in proximal tubular epithelial cells

**Fig. 3:**
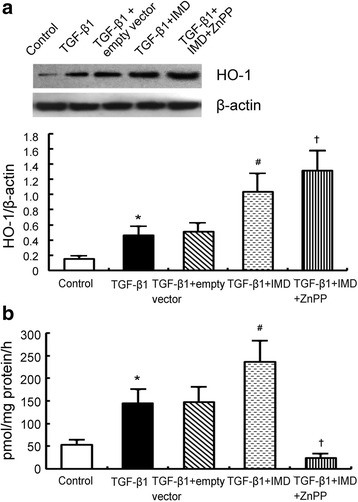
IMD induces HO-1 expression and HO activity in TGF-β1-stimulated proximal tubular epithelial cells NRK-52E. **a** Respective Western blot analysis and quantitation of HO-1 protein levels in TGF-β1-stimulated NRK-52E. **b** HO activity in TGF-β1-stimulated NRK-52E. Data in *bar graphs* are means ± SD, *n* = 6. ∗ *P* < 0.05 vs. sham control group; # *P* < 0.05 vs. UUO group; † *P* < 0.05 vs. UUO + IMD group

We investigated the effect of IMD on HO-1 expression and HO activity in rhTGF-β1-stimulated rat proximal tubular epithelial cell line NRK-52E. Our results showed that HO-1 expression and HO activity were induced by rhTGF-β1 administration. Overexpression of IMD by gene transfer further increased HO-1 expression and HO activity. ZnPP up-regulated HO-1 expression but reduced HO activity. As a control, empty plasmid had little effect on HO activity (Fig. 3).

### IMD reduces renal fibrosis in obstructed kidneys by induction of HO-1

**Fig. 4:**
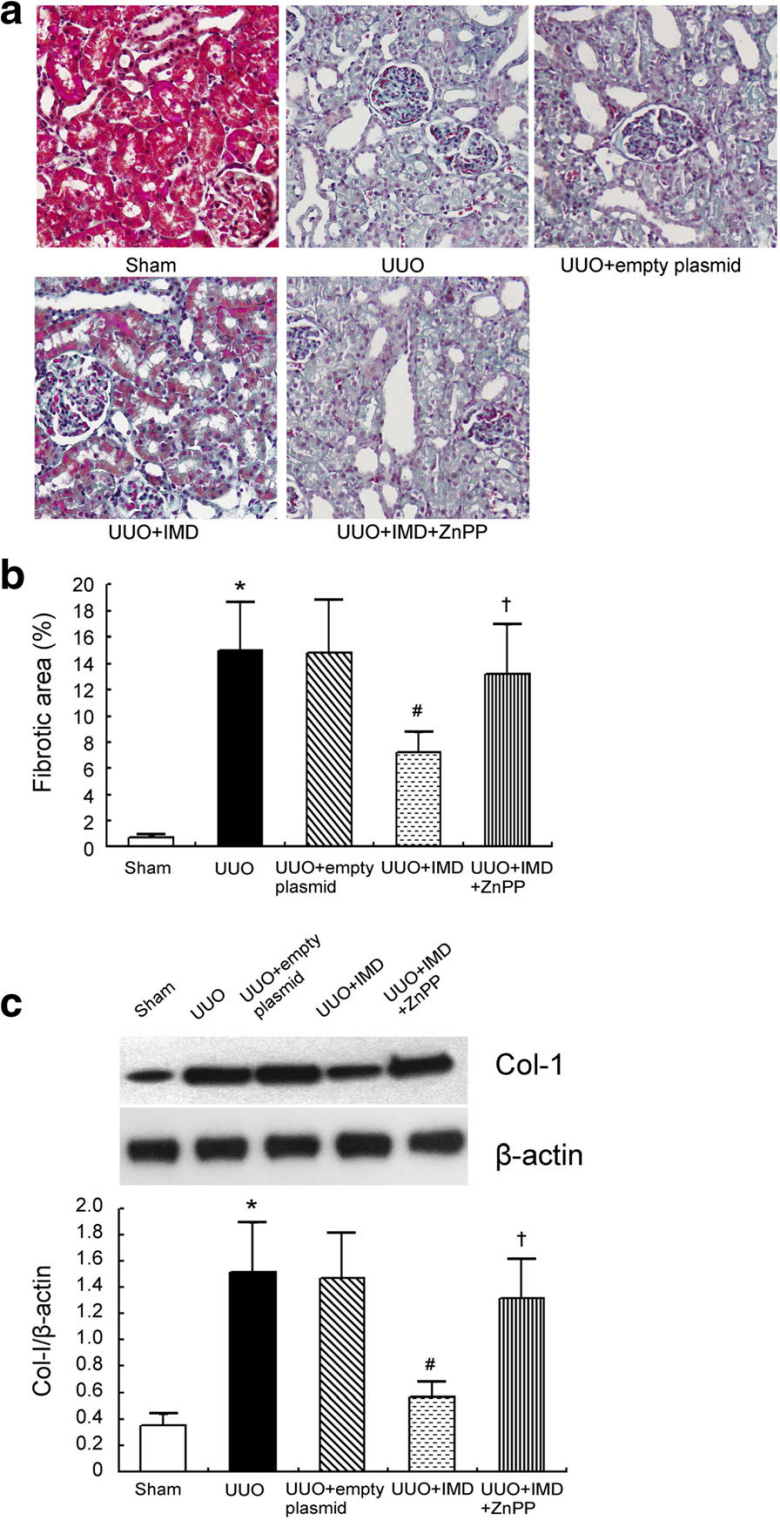
IMD inhibits renal fibrosis induced by UUO by induction of HO-1. **a** Representative Masson’s trichrome stained sections of obstructed kidney of UUO rats. Original magnification, ×200. **b** Semiquantitative score of tubulointerstitial fibrosis of obstructed kidneys. **c** Respective Western blot analysis and quantitation of Col-1 expression in the obstructed kidney of UUO rats. Data in *bar graphs* are means ± SD, *n* = 6.∗ *P* < 0.05 vs. sham control group; # *P* < 0.05 vs. UUO group; † *P* < 0.05 vs. UUO + IMD group

Tubulointerstitial fibrosis was assessed by Masson’s trichrome staining for collagen as we previously described [7]. No changes were detected in the sham group. We showed that tubulointerstitial fibrosis was produced in the obstructed kidney of UUO rats 7 days after operation compared with sham controls. By contrast, IMD gene delivery significantly ameliorated UUO-induced tubulointerstitial fibrosis in the obstructed kidneys. To ascertain the anti-fibrotic role of IMD could be afforded by induction of HO-1, ZnPP, a known inhibitor of HO enzymatic activity, was used. As expected, the suppression effect of tubulointerstitial fibrosis by IMD was blocked by ZnPP. Increased renal fibrosis in UUO was confirmed by Col-I, a major ECM protein, expression. Western blot of Col-I yielded significant increase in Col-I expression in the untreated UUO rats compared with the sham. IMD treatment markedly inhibited the up-regulation of Col-I. ZnPP administration abrogated the protective effect of IMD on Col-I expression. These data demonstrate that the overexpression of IMD ameliorates renal interstitial fibrosis by induction of HO-1 (Fig. 4).

### IMD inhibits inflammation in the obstructed kidneys by induction of HO-1

**Fig. 5:**
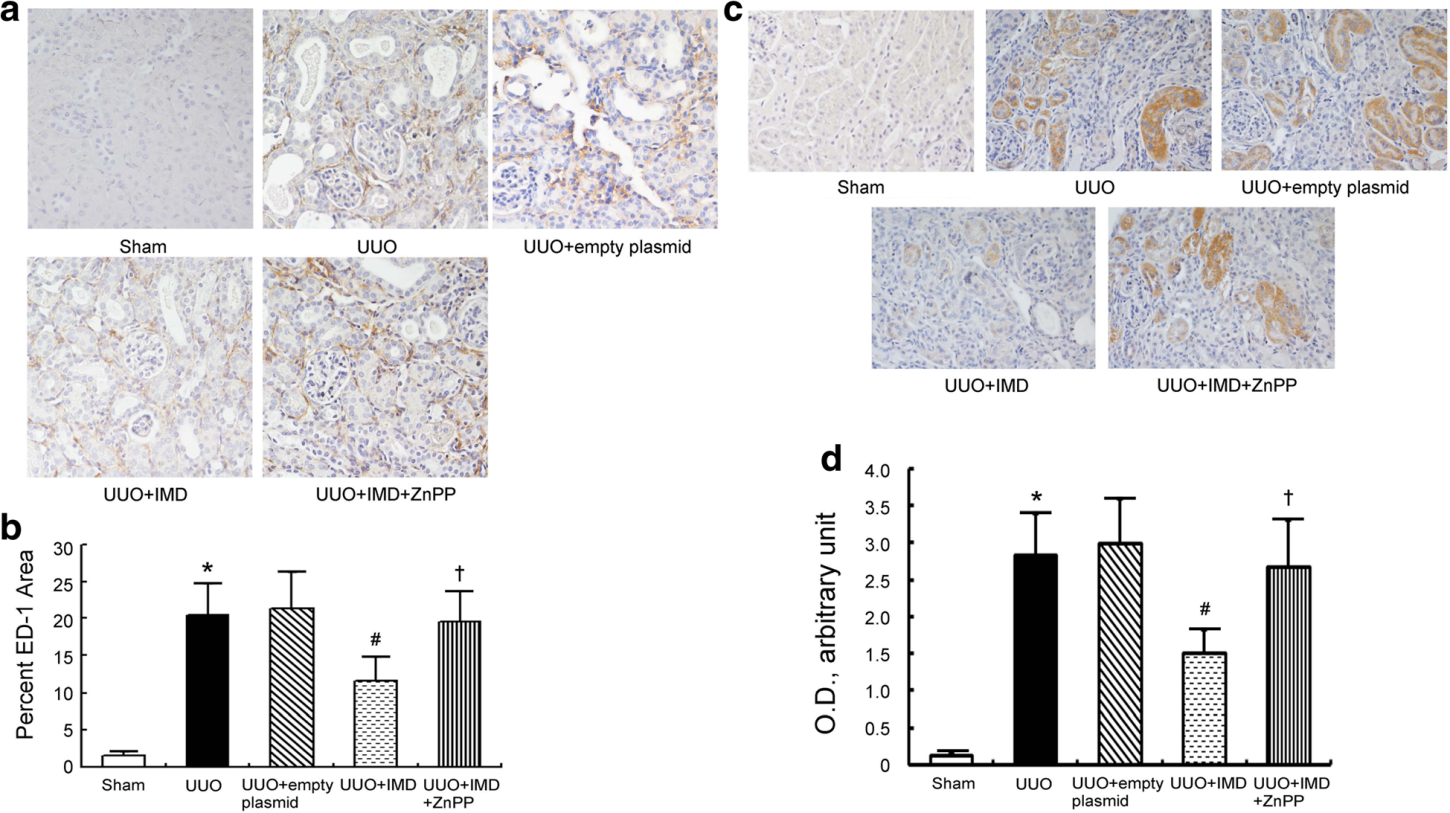
IMD decreases UUO-induced inflammation by induction of HO-1. **a** Representative immunohistochemical staining for the infiltration of ED-1-positive cells. Original magnification, ×400. **b** Quantitative analysis of ED-1-positive cells. **c** Representative immunohistochemical staining for IL-6 expression. Original magnification, ×400. **d** Semiquantitative evaluation of IL-6 expression. Data in *bar graphs* are means ± SD, *n* = 6. ∗ *P* < 0.05 vs. sham control group; # *P* < 0.05 vs. UUO group; † *P* < 0.05 vs. UUO + IMD group

To investigate whether IMD could inhibit renal inflammation, we evaluated macrophage infiltration and IL-6 expression in the obstructed kidneys of UUO rats. Staining for ED-1 revealed the degree of interstitial macrophage infiltration. Few ED-1-positive cells were observed in the interstitium of sham-operated kidneys. The number of ED-1-positive cells in the obstructed kidneys from UUO rats markedly increased, mainly in the periglomerular and peritubular areas. IMD gene transfer significantly inhibited macrophages infiltration, which could subsequently be blocked by the HO-1 inhibitor ZnPP (Fig. 5a and b). Pro-inflammatory cytokine IL-6 expression was elevated in the obstructed kidneys after UUO operation as compared with the sham controls, which was inhibited by IMD gene delivery. ZnPP attenuated the protective effect of IMD on IL-6 expression (Fig. 5c and d).

### UUO-induced tubular epithelial cells EMT is inhibited by IMD via induction of HO-1

**Fig. 6:**
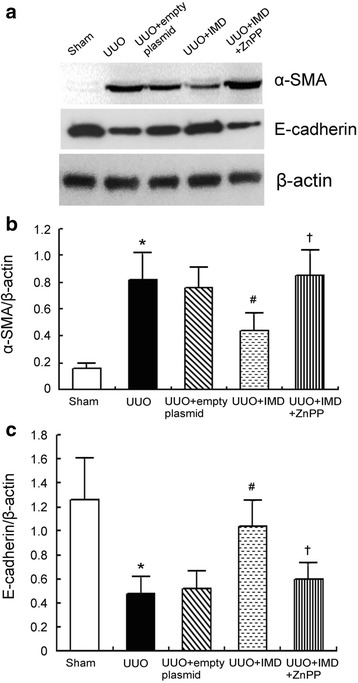
IMD inhibits UUO induced EMT in vivo by induction of HO-1. **a** Representative α-SMA and E-cadherin protein expression measured by Western blot in rats. **b** Densitometric quantifications of band intensities from Western blot for α-SMA/β-actin in the obstructed of UUO rats. **c** Densitometric quantifications of band intensities from Western blot for E-cadherin/β-actin in the obstructed of UUO rats. Data in *bar graphs* are means ± SD, *n* = 6. ∗ *P* < 0.05 vs. sham control group; # *P* < 0.05 vs. UUO group; † *P* < 0.05 vs. UUO + IMD group

For determination if IMD could inhibit UUO-induced EMT, we examined α-SMA, a mesenchymal marker, and E-cadherin, an epithelial marker, expressions by Western blot analysis. We showed that α-SMA expression up-regulated significantly, while E-cadherin down-regulated markedly in the obstructed kidneys of untreated UUO rats. IMD inhibited UUO-induced increase of α-SMA expression and decrease of E-cadherin in the obstructed kidney. Inhibition of HO-1 enzyme activity by ZnPP reversed the effects of IMD (Fig. 6).

### TGF-β1-mediated proximal tubular epithelial cells EMT is inhibited by IMD via induction of HO-1

**Fig. 7:**
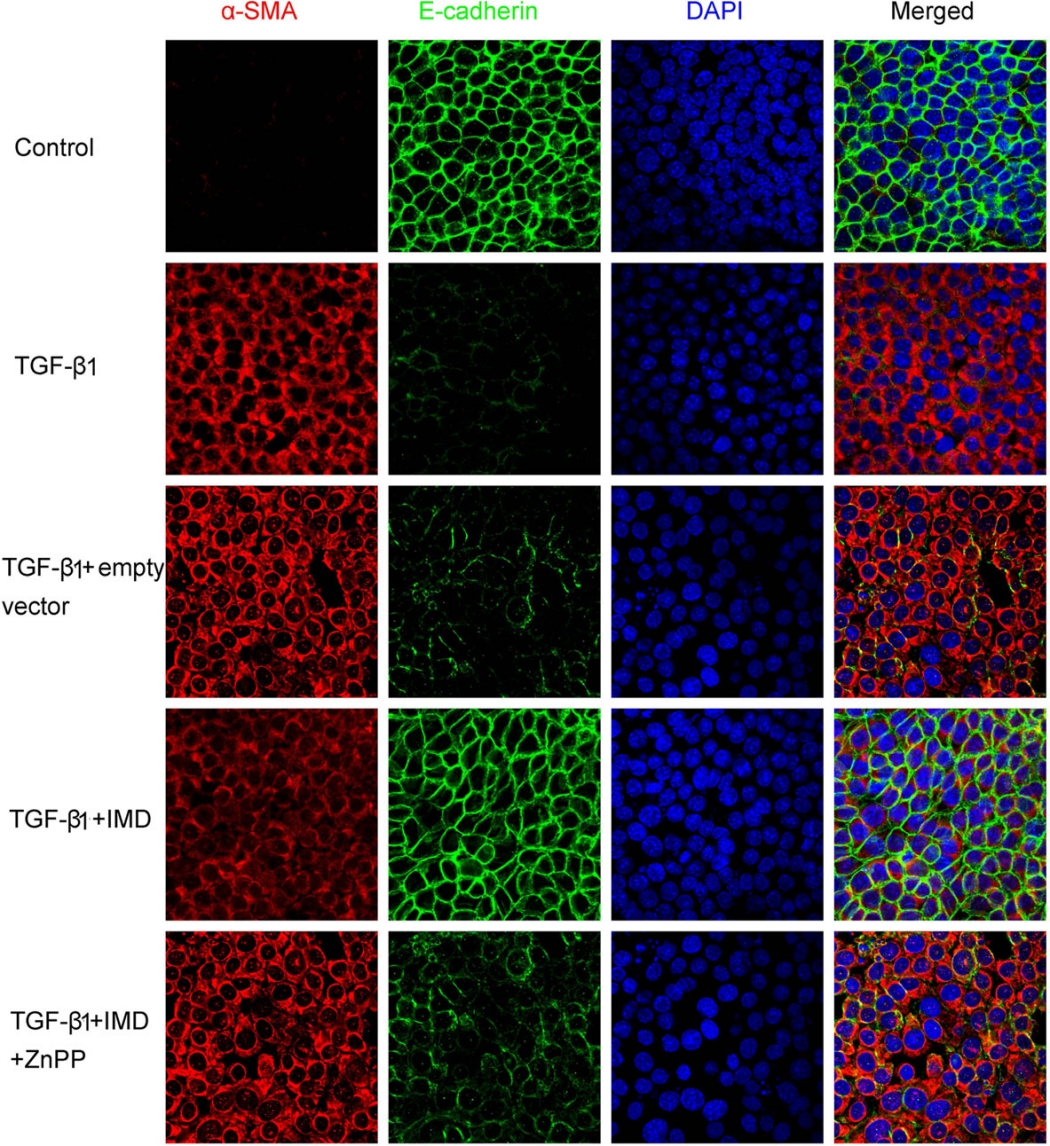
IMD inhibits TGF-β1-mediated proximal tubular epithelial cells NRK-52E EMT by induction of HO-1. Representative immunofluorescence staining of α-SMA (*red*) and E-cadherin (*green*) with nuclear stain (DAPI, *blue*) in NRK-52E. Original magnification, ×400

To confirm the effect of IMD on EMT, we examined α-SMA/E-cadherin double staining in rat proximal tubular epithelial cell line NRK-52E by immunofluorescence assay. In response to rhTGF-β1, E-cadherin expression decreased, while α-SMA increased. IMD increased E-cadherin expression and suppressed α-SMA expression significantly. The protective effect of IMD was abolished by the addition of ZnPP (Fig. 7).

## Discussion

Tubulointerstitial fibrosis is a common pathway of all CKD leading to ESRD. Furthermore, it remains the best predictor of disease progression [20]. In the present study, we analyzed the protective effect of IMD on renal fibrosis in rat UUO model, a well-established in vivo model of renal fibrosis. Our results showed that IMD reduced parameters of fibrosis such as elevated collagen deposition measured by Masson’s trichrome staining, and Col-I production. In addition, IMD inhibited macrophages infiltration, pro-inflammatory cytokine IL-6 expression and EMT in UUO-induced kidneys. In vitro studies demonstrated that TGF-β1-stimulated EMT in proximal tubular epithelial cells was reduced by IMD. Furthermore, all the above beneficial effects of IMD were abolished by inhibition of HO-1 enzyme activity with ZnPP.

It is commonly accepted that ROS have been implicated in the pathophysiology of renal fibrosis [21]. Our previous studies demonstrated that IMD attenuated renal fibrosis by inhibition of oxidative stress [7] and the precise mechanisms warrant further studies. HO is a microsomal enzyme that exists in two isoforms: the inducible HO-1 and the constitutive HO-2. HO-1 is a stress response protein with antioxidative properties [22]. It is reported that induction of HO-1 is an adaptive and protective response against renal fibrosis [17]. Whether IMD has any effect on HO-1 expression and HO activity has not been investigated yet. In this study, we observed a significant amplification of HO-1 mRNA and protein as well as HO activity in IMD-treated animals (Fig. 2a–d). Immunohistology reveals that IMD-induced HO-1 protein expressions were mainly in tubular epithelial cells (Fig. 2c). We reached the same results in TGF-β1-stimulated tubular cells (Fig. 3). Importantly, this upregulation of HO-1 and induction of HO activity by IMD was paralleled with suppression of tubulointerstitial fibrosis (Fig. 4).

To verify if the anti-fibrotic effect of IMD is achieved by induction of HO-1, we used ZnPP. It has been reported that ZnPP inhibited HO-1 enzyme activity, although it increased HO-1 protein and mRNA expression [23]. Although HO-2 activity is also inhibited by ZnPP, the constitutive nature of HO-2 makes it less attractive as a drug target [10]. In line with previous reports, our results indicated that ZnPP markedly decreased HO activity in obstructed kidney and cultured tubular cells. HO activity in contralateral kidney of UUO rat was also inhibited by ZnPP, indicating that the effect of ZnPP on HO activity was systemic. We showed that inhibition of HO enzyme activity abrogated the protective effect of IMD on interstitial fibrosis. Thus, the antifibrotic effect of IMD in our model is dependent on induction of HO-1.

A number of evidence demonstrated that oxidative stress could trigger the inflammatory response in renal fibrosis [24]. Inflammation plays an important role in the development and progression of renal fibrosis [25]. Macrophages, a major type of inflammatory cell, are recruited during tubulointerstitial fibrosis [26, 27]. IL-6 is a well-known pro-inflammatory cytokine that is also correlated with increased fibrosis [28]. In this study, we showed the number of macrophages and expression of IL-6 in the obstructed kidneys of UUO rats was significantly higher than that of the sham-operated rats. Although the recruitment of macrophages may contribute to kidney repair at the early stages, this repair can ultimately cause renal fibrosis [27, 29]. In our study, in line with the infiltration of macrophages and up-regulation of IL-6, renal fibrosis is associated enhanced (Fig. 4). IMD overexpression obviously inhibited the infiltration of macrophages and IL-6 expression following UUO, which effect could subsequently be blocked by the HO-1 inhibitor ZnPP. This indicates that the protection against macrophage infiltration and IL-6 production afforded by IMD was due to induction of HO-1.

EMT plays a pivotal role in the pathogenesis of renal tubulointerstitial fibrosis [30]. EMT is recognized as a loss of epithelial cell phenotype, decrease of intercellular epithelial adhesion molecules, such as E-cadherin, with a concomitant development of mesenchymal phenotype, including expression of α-SMA [31]. In this study, we showed that α-SMA expression significantly increased, while E-cadherin expression markedly decreased, after UUO operation, indicating extensive EMT (Fig. 6). It has been suggested that EMT is mediated, at least in part, through ROS [32]. Thus, IMD, through its antioxidant properties, may reduce EMT. Our results demonstrated that IMD gene delivery attenuated UUO-induced EMT, which was abolished by HO-1 activity inhibition.

For further clarification of the effects of IMD on TGF-β1-mediated EMT in proximal tubules, in vitro studies were performed to eliminate the confounding effects of infiltrating cells. These in vitro studies, using rat proximal tubular cells (NRK-52E), indicated that IMD inhibited TGF-β1-mediated EMT as indicated by up-regulation of E-cadherin and down-regulation of α-SMA measured by immunofluorescence (Fig. 7). ZnPP abrogated the beneficial effects of IMD on EMT. These results, along with the in vivo evidence, demonstrate that IMD inhibits EMT by induction of HO-1.

Although our current study provided significant results, there are still some limitations. The primary one is that we did not use HO-1-deficient mice or HO-1-deficient tubular cells to investigate the effect of IMD on renal fibrosis. ZnPP is commonly used as a HO-1 inhibitor [15], however, its specificity is still a matter of debate [33, 34]. The second limitation is the use of UUO as an animal model CKD. Although UUO is a very well-accepted renal fibrosis model, some experts criticized it as a model of CKD because of the compensation by the normal contralateral kidney that precludes meaningful renal functional measures [35]. More CKD models that provide us with an opportunity to include renal functional study end-points together with quantitative measures of fibrosis severity are needed. The last one is we didn’t investigate the transcription pathways by which IMD activates HO-1. Examining some transcription factors involved in HO-1 modulation are required to provide further mechanistic insight into the mechanism of IMD induced HO-1 activation pathway.

## Conclusion

In summary, our study demonstrates that IMD gene transfer significantly attenuates renal fibrosis in parallel with the blockade of inflammation and EMT of tubular cell, and that this is associated with an increased HO-1 expression and HO activity. Blockade of HO enzyme activity by a specific inhibitor completely abrogates the effects of IMD, indicating that the beneficial effects of IMD are achieved by HO-1 induction. The results indicate that induction of HO-1 by IMD may be a potential therapeutic strategy to prevent renal fibrosis in patients with CKD.

## Additional file

Additional file 1: Figure S1. HO activity in the contralateral kidneys of UUO rats. † *P* < 0.05 vs. UUO + IMD group. (PDF 123 kb)

## Abbreviations

ADM: Adrenomedullin
cDNA: Complementary DNA
CKD: Chronic kidney disease
Col-I: Collagen I
ECM: Extracellular matrix
EMT: Epithelial-mesenchymal transition
ESRD: End stage renal diseases
HO: Heme oxygenase
IMD: Intermedin
ROS: Reactive oxygen species
SD: Standard deviation
UUO: Unilateral ureteral obstruction
ZnPP: Zinc protoporphyrin
α-SMA: α-smooth muscle actin
