# Supplementary figures and images for: Intermedin attenuates renal fibrosis by induction of heme oxygenase-1 in rats with unilateral ureteral obstruction
Source: BMC Nephrol. 2017 Jul 11;18:232. doi: 10.1186/s12882-017-0659-6 (PMC5505135; doi:10.1186/s12882-017-0659-6)

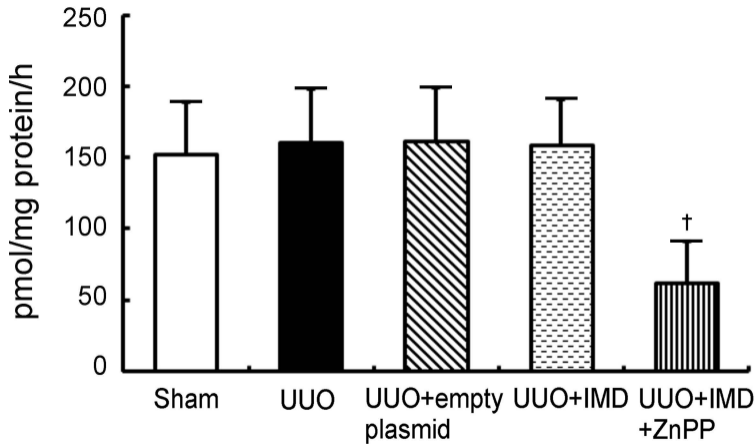

Supplement: Additional file 1: Figure S1. — HO activity in the contralateral kidneys of UUO rats. † P < 0.05 vs. UUO + IMD group. (PDF 123 kb) [file 12882_2017_659_MOESM1_ESM.pdf]
